# Knowledge of sexually transmitted infections and its associated factors among polytechnic college students in Southwest Ethiopia

**DOI:** 10.11604/pamj.2020.37.68.22718

**Published:** 2020-09-17

**Authors:** Tadesse Nigussie, Tewodros Yosef

**Affiliations:** 1 Department of Public Health, College of Medicine and Health Sciences, Mizan-Tepi University, Mizan Teferi, Ethiopia

**Keywords:** Knowledge, sexually transmitted infections, polytechnic college, Ethiopia

## Abstract

**Introduction:**

sexually transmitted infections (STIs) are infections that are transmitted from one person to another through sexual contact, and most of them are easily preventable and treatable. Global trends in STIs have increased. Sub-Saharan Africa carries a high burden of STIs, including HIV. Knowledge about STIs is very significant for preventing the adverse outcomes of young adult reproductive health. Therefore, this study aimed to assess the knowledge of sexually transmitted diseases and its associated factors among polytechnic college students in Southwest Ethiopia.

**Methods:**

a cross-sectional study was conducted among 453 randomly selected students at Mizan-Aman polytechnic college in Southwest Ethiopia from April 1^st^ to 30^th^, 2018. The data were collected through self-administered structured and pre-tested questionnaire. The collected data were entered using EPI-DATA version 4.2.0.0, and analyzed using SPSS version 20 statistical software.

**Results:**

of the 453 participants, 177 (39.1%) had good knowledge about STIs. The study also found that being male (AOR = 1.72, 95% CI [1.12-2.86], P = 0.025), increased year of study (AOR = 3.65, 95% CI [1.69-8.43], P = 0.002), having one or no sexual partner (AOR = 1.53, 95% CI [1.35-3.56], P = 0.005), and source of information from mass media (Television/Radio) (AOR = 2.76, 95% CI [1.78-4.56], 0.013) were factors that associated with an increased level of knowledge about STIs.

**Conclusion:**

the proportion of good knowledge about sexually transmitted infections was substantially low. Therefore, strengthening information, education, and communication (IEC) on the issue using health clubs and mass media (Television/Radio) is highly recommended. In addition, inculcating the sexual and reproductive health course in the educational curriculum plays a paramount importance.

## Introduction

Sexually transmitted infections (STIs) are infections that are transmitted from one person to another through sexual contact, and most of them are easily preventable and treatable [1, 2]. STIs represent a huge burden of disease worldwide with an annual incidence of about 333 million cases and have harmful effects on sexual health or reproduction [3-5]. Global trends in STIs have increased. Sub-Saharan Africa carries a high burden of STIs, contributing to more than 70% of the universal burden of infection [6]. Sexually transmitted infections are a major health problem that affects mostly young people in developing as well as developed countries [7] due to sexual experimentation occurring at this age [8]. Young adults are most vulnerable to infection because they engage in risky practices due to a lack of adequate knowledge of STIs [9, 10]. Knowledge of STIs is very significant for preventing adverse outcomes of young adult reproductive health [11, 12]. Insufficient knowledge about sexually transmitted infections is the major barrier to successfully prevent infection among young adult populations [13]. Since the lack of knowledge of STIs may lead to a delay in treatment [5], it may complicate the infection process. The health-seeking behavior of STIs may largely depend on knowledge about STIs [14]. Several studies have been conducted worldwide regarding knowledge about STIs and reported that 74.7% in India [15], 92.4% in Nigeria [16], 89.9% in Brazil [17], 98% in Tanzania [18], 88.5% in Jimma, Ethiopia [19], 79% in Dhaka, Bangladesh [20], 86.6% in Malaysia [21], 74% medical and 61.6% non-medical university students in Pakistan [22], 83.1% in Turkey [23], 68.3% in Klang Valley, Malaysia [24], 27% in Udupi Taluk, India [25], and 70.1% in northern Cape Province, South Africa [26] of the respondents had good knowledge of STIs. The factors that influence knowledge of STIs are diverse and include age, sex, residence, marital status, academic year, and acquiring information from friends/internet and mass media [5, 18, 27-34].

In sub-Saharan Africa, comprehensive accurate knowledge about STIs remains low in most countries [35], especially in Ethiopia, knowledge of STIs is very low. According to EDHS 2016, 20% of women and 38% of men have comprehensive knowledge about STIs transmission and prevention [36]. In Ethiopia, young adults are frequently involved in risky sexual behavior [37, 38], which predispose them to a multiplicity of sexual and reproductive health problems. Improving STIs awareness through inculcating sexual and reproductive health into the educational curriculum to prevent and control STIs plays paramount importance [26]. Despite the fact that the prevalence of STIs is high in Ethiopia [39], there is no sufficient shreds of evidence that showed knowledge about STIs among adolescents and youths in the study area. To design appropriate intervention for improving the knowledge of STIs among young adults, the availability of sufficient data on the issue is of paramount importance. Therefore, this study aimed to assess the knowledge of sexually transmitted diseases and its associated factors among polytechnic college students in Southwest Ethiopia.

## Methods

**Study design, setting, and period:** a cross-sectional study was conducted at Mizan-Aman polytechnic college (MAPtC) students from April 1^st^ to 30^th^, 2018. MAPtC is found at 585 km southwest of Addis Ababa, the capital city of Ethiopia. The college teaches students in ten departments, with five/four levels for each department. The departments were Garment and Textile, Automotive, Road construction, Water and Sanitation, Information communication technology, Building electrical installation, Electrotechnology, Masonry construction, General metal fabrication, and Surveying technology.

**Populations:** the source of the population was all regular students at the polytechnic college, who were attending their class during the study period. The study population was randomly selected students who studied at the polytechnic college during the study period.

**Sample size determination and sampling method:** the sample size was determined using a single population proportion formula. With an input of expected proportion of knowledge about STIs in Chercher, Ethiopia (17.5%) [13], 5% margin of error, 95% confidence interval, 10% for non-response compensation, and a design effect of 2. The final computed sample size was 489. A two-stage stratified random sampling technique was used to select 489 regular students. In polytechnic college, there were ten departments with five/four levels for each department. The departments were stratified based on levels (level I-V). For each level, the sample size is proportionally allocated. The potential participants were selected using systematic random sampling.

**Data collection instrument and procedures:** data were collected through self-administered structured and pre-tested questionnaire. The questionnaire was composed of three sections (socio-demographic factors, knowledge regarding STIs including HIV (including 4 questions on type, symptoms, mode of transmission, and prevention of STIs with a total of 18 items) and behavioral factors). The questionnaire was developed by reviewing relevant literature in English, then translated it into the local language, and retranslated back it into English to check the consistency by an independent translator. The training was given to data collectors and supervisors concerning the objective and process of data collection and to discuss the presence of an ambiguous question in the questionnaire.

**Study variables:** the dependent variable was knowledge of sexually transmitted infections. The independent variables were age, sex, residence, marital status, religion, academic year, drinking alcohol, smoking cigarette, condom use, watching pornography, number of sexual partners, and source of acquiring information.

**Operational definitions:** good knowledge was defined as those who scored the mean and above value of knowledge-related questions [14]. Poor knowledge was defined as those who scored below the mean value of knowledge-related questions [14]. Multiple sexual partners are defined as the behavior of a person with two or more sexual partners [37].

**Data processing and analysis:** the collected data were entered into EPI-Data version 4.2.0.0, and analyzed using SPSS version 20 software for windows. The results are presented in tables and numerical summery measures (mean and standard deviation). A binary logistic regression analysis was used to look for the association between outcome and independent variables and dependent variables. The independent variables with a p-value of less than 0.25 in the bivariate logistic regression were included in the multivariable logistic regression. Finally, variables in the multivariable logistic regression with a p-value < 0.05 were considered as significantly associated with the outcome variable. The Hosmer-Lemeshow goodness-of-fit test indicated (P = 0.756) that the model was good enough to fit the data well.

**Ethical consideration:** before the actual data collection, a permission letter was obtained from Mizan-Tepi University to Mizan Aman Polytechnic College. All study participants were informed about the purpose of the study, their right to deny participation, anonymity, and confidentiality of the information. Written informed consent was also obtained before participation in the study.

## Results

**Socio-demographic characteristics:** of the 489, 453 students filled the questionnaires making a response rate of 92.6%. The majority of the respondents were male (53.6%), single (88.1%), and orthodox religious followers (54.7%). More than half (57.6%) of the respondents were from urban. The mean age of the respondents was 20 (±2.02 SD) years, ranging from 18 to 30 years (Table 1).

**Table 1:** socio-demographic characteristics of the respondents at MAPtC in Southwest Ethiopia

Variables	Categories	Frequency	Percent
**Sex**	Male	243	53.6
	Female	210	46.4
**Age**	< 20 years	217	47.9
	≥ 20 years	236	52.1
**Religion**	Orthodox	248	54.7
	Protestant	144	31.8
	Muslim	61	13.5
**Marital status**	Single	399	88.1
	Married	46	10.2
	Divorced	8	1.7
**Residence**	Rural	192	42.4
	Urban	261	57.6
**Year of the study**	First-year	51	11.3
	Second-year	98	21.6
	Third-year	180	39.7
	Fourth-year	124	27.4

**Knowledge of STIs and source of information for STIs:** of the 453 respondents, 216 (47.7%) were mentioned sexual intercourse as modes of STIs transmission. One-hundred fifty (33.1%) responded condoms used to prevent STIs and 10 (2.2%) had misconceptions (contraceptive pills) about STIs prevention (Table 2). Of the 453, 177 (39.1%) had good knowledge of sexually transmitted infections. Of the 177 respondents with good knowledge about sexually transmitted infections, 103 (58.2%) were male and 74 (41.8%) were female students. One hundred fifty-four (34%) had a source of information from health professionals followed by 136 (30%), 95 (21%), 40 (8.8%) and 28 (6.2%) had a source of information from mass media (Television/Radio), friends, parents, and newspapers/books respectively.

**Table 2:** knowledge regarding STIs among respondents at MAPtC in Southwest Ethiopia

Variables	Categories	Yes, n (%)	No, n (%)
**Known STI types**	Syphilis	150(33.1)	303(66.9)
	Gonorrhea	127(28)	326(72)
	Chancroid	54(11.9)	399(88.1)
	HIV/AIDS	247(54.5)	206(45.5)
	Hepatitis B&C	38(8.4)	415(91.6)
**Known STI symptoms**	Genital ulcer	143(31.6)	310(68.4)
	Genital discharge	74(16.3)	379(83.7)
	Burning and/ pain on urination	144(31.8)	309(68.2)
	Genital swelling	43(9.5)	410(90.5)
**Modes of STI transmission**	Sexual intercourse	216(47.7)	237(52.3)
	Breastfeeding	35(7.7)	418(92.3)
	Blood transfusion	76(16.8)	377(83.2)
	Sharing contaminated materials	55(12.1)	398(87.9)
**Methods of STI prevention**	Abstinence	122(26.9)	331(73.1)
	Being faithful	65(14.4)	388(85.6)
	Condom use	150(33.1)	303(66.9)
	Not sharing sharp materials	45(9.9)	408(90.1)
	Contraceptive pills	10(2.2)	443(97.8)

**Behavioral profiles:** forty-seven (10.4%) respondents were cigarette smokers. Nearly one-fourth (23.6%) and 172 (38%) of the respondents were alcohol drinkers and watching pornography at least once a time in lifetime respectively. Of the 180 sexually active, 119 (66.1%) and 82 (45.6%) used condoms in their last sexual intercourse and had multiple sexual partners, respectively, (Table 3).

**Table 3:** behavioral profiles of the respondents at MAPtC in Southwest Ethiopia

Variables	Categories	Frequency	Percent
**Cigarette smoking**	Yes	47	10.4
	No	406	89.6
**Drinking alcohol**	Yes	107	23.6
	No	346	76.4
**Condom utilization**	Yes	119	66.1
	No	61	33.9
**Watching pornography**	Yes	172	38
	No	281	62
**Multiple sexual partners**	No	98	54.4
	Yes	82	45.6

**Factors associated with knowledge of STIs:** bivariate analysis was done for potentially expected associated factors. Independent variables found statistically significant at P < 0.25 in the bivariate analysis were included in the multivariable binary logistic regression model. Finally, sex, academic year, number of sexual partners, and acquiring information from mass media (Television/Radio) were found to be significantly associated with good knowledge of STIs (Table 4).

**Table 4:** factors associated with good knowledge of sexually transmitted infections of the respondents at MAPtC in Southwest Ethiopia

Variables	Categories	Knowledge of STIs		COR (95% CI)	AOR (95% CI)	P-value
		Good	Poor			
**Age group**	< 20 years	90	127	1	1	
	≥ 20 years	87	149	0.82(0.41-1.12)^*^	0.58(0.45-1.46)	0.053
**Sex**	Male	103	140	1.35(1.29-3.36)^**^	1.72(1.12-2.86)	**0.025**
	Female	74	136	1	1	
**Year of study**	First year	13	38	1	1	
	Second year	31	67	1.35(0.56-2.62)^*^	1.45(0.87-4.01)	0.256
	Third year	63	117	1.57(0.79-3.37)^*^	1.84(0.88-4.24)	0.156
	Fourth year	70	54	3.79(1.54-7.11)^**^	3.65(1.69-8.43)	**0.002**
**Multiple sexual partners**	No	77	21	1.26(1.12-2.49)^**^	1.53(1.35-3.56)	**0.005**
	Yes	61	21	1	1	
**STI information from Health professionals**	Yes	103	125	1.68(1.15-2.46)^**^	1.75(0.89-2.85)	0.062
	No	74	151	1	1	
**STI information from media (TV/Radio)**	Yes	100	91	2.64(1.79-3.90)^**^	2.76(1.78-4.56)	**0.013**
	No	77	185	1	1	

CI = Confidence Interval, COR = Crude odds ratio, AOR = Adjusted odds ratio, ^*^ = significant at a p-value < 0.25, ^**^ = significant at a p-value < 0.05

## Discussion

Several studies have revealed that sexually transmitted infections are the cause of the multiplicity of complications and result in poor sexual and reproductive health due to delays in treatment as a result of a lack of knowledge about STIs [5, 40]. Health-seeking behavior may largely depend on knowledge about STIs [14]. Based on the above scenario, we aimed to assess the knowledge of sexually transmitted diseases and its associated factors among polytechnic college students in Southwest Ethiopia. The proportion of good knowledge about sexually transmitted infections was 39.1% (34.6% - 43.6%). This finding was higher than 27% in Udupi Taluk, India [25]. However, lower than 45.4% in Gondar, Ethiopia [35], 68.3% in the Klang Valley, Malaysia [24], 70.1% in northern Cape Province, South Africa [26], 74.7% in urban slums of Jorhat District, India [15], 79% in Dhaka, Bangladesh [20], 86.6% in Malaysia [21], 89.9% in Brazil [17], 92.4% in Nigeria [16], and 98% in Dar es Salaam, Tanzania [18]. The variation observed compared to other studies could be due to the differences in methodology, sample size, and operational definition used. Besides the sociocultural, socioeconomic, and behavioral characteristics of the study participants may play a great role in the variation observed.

In this study, being male was associated with good knowledge about STIs. This finding was supported by a study conducted in Addis Ababa, Ethiopia [10]. However, a study conducted in Portugal revealed that women had greater knowledge than men [32]. Another study revealed no variation was found between gender and the level of knowledge about sexually transmitted infections [29]. Respondents with increased academic years were associated with having good knowledge about STIs. This study was consistent with a study conducted in Nepal [21, 34]. This could be because of the higher level of education related to more knowledge about STIs. Among sexually active, 98 (54.4%) of the respondents had fewer than two sexual partners (means one or no). Respondents with fewer than two sexual partners were significantly associated with good knowledge about STIs. Those with one and no sexual partners due to the fear of the diseases resulted from their knowledge of the symptoms and health consequences of STIs than those with having multiple sexual partners. However, the result was inconsistent with a study conducted in Sweden, which revealed that the experience of many sexual partners and having a history of STIs were associated with a higher level of knowledge [41] and in southern Gondar Ethiopia, the seroprevalence of syphilis was significantly associated with women who have multiple sexual partners [42]. This variation could be explained by the differences in sociocultural and behavioral characteristics.

Mass media (Television/Radio) is an important method, that is used to create awareness for a large audience on a certain issue. Thus, mass media (Television/Radio) exposure was positively associated with good knowledge about a specific issue. Of the 177 respondents with good knowledge about STIs, 100 (56.5%) of the respondents had exposure to mass media (Television/Radio). Getting information from mass media (Television/Radio) was significantly associated with having good knowledge about STIs. This finding is consistent with previous studies done in Tanzania, Nepal, and Vietnam [5, 18, 34]. The authors acknowledge some limitation of the study, the nature of the study design may not show a cause-and-effect relationship. It was also difficult to ascertain which comes first between the association of attitude toward condom use and knowledge about sexually transmitted infections.

## Conclusion

The proportion of good knowledge about sexually transmitted infections was substantially low. Therefore, strengthening information, education, and communication (IEC) on the issue using health clubs and mass media (Television/Radio) is highly recommended. In addition, inculcating the sexual and reproductive health course in the educational curriculum plays a paramount importance.

### What is known about this topic

- Sexually transmitted infections are a major health problem that affects mostly young people in developing as well as developed countries;
- Young adults are most vulnerable to infection because they engage in risky practices due to a lack of adequate knowledge of STIs; knowledge of STIs is very significant for preventing adverse outcomes of young adult reproductive health;
- In sub-Saharan Africa, comprehensive accurate knowledge of STIs remains low in most countries, especially in Ethiopia, knowledge of STIs is very low.

### What this study adds

- The proportion of good knowledge of sexually transmitted infections was substantially low;
- The study found that sex, academic year, number of sexual partners, and acquiring information from mass media (Television/Radio) were found to be significantly associated with good knowledge of sexually transmitted infections;
- Therefore, it is very important to inculcate the sexual and reproductive health course in the educational curriculum to increase students’ knowledge level of sexually transmitted infections.
